# Influence of Entrepreneurial Orientation on Venture Capitalists' Initial Trust

**DOI:** 10.3389/fpsyg.2021.633771

**Published:** 2021-04-01

**Authors:** Hongtao Yang, Lei Zhang, Yenchun Jim Wu, Hangyu Shi, Shuting Xie

**Affiliations:** ^1^School of Business Administration, Huaqiao University, Quanzhou, China; ^2^Graduate Institute of Global Business and Strategy, National Taiwan Normal University, Taipei, Taiwan; ^3^College of Management, National Taipei University of Education, Taipei, Taiwan

**Keywords:** entrepreneurial orientation, entrepreneurial intention, initial trust, impression management strategies, perceptions of hypocrisy

## Abstract

The effectiveness of trust has been extensively investigated in entrepreneurship studies. However, compared to the outcomes of trust, we still lack knowledge about the mechanisms underlying venture capitalists' initial trust in entrepreneurs. Drawing from signal theory and impression management theory, this study explores an impression management motivational explanation for the influencing factors of venture capitalists' initial trust. An empirical test is based on 202 valid questionnaires from venture capitalists, and the results indicate that the signal of five dimensions of entrepreneurial orientation has a significant impact on the initial trust of venture capitalists and that a signal of entrepreneurial orientation of perseverance or passion positively influences venture capitalists' initial trust through acquired impression management strategies, while a signal of entrepreneurial orientation of risk-taking, innovation, or proactivity positively affects the initial trust of venture capitalists through defensive impression management strategies. The perceptions of entrepreneurs' hypocrisy by venture capitalists negatively moderate the relationship between acquired impression management strategies and the initial trust of venture capitalists and negatively moderate the relationship between defensive impression management strategies and the initial trust of venture capitalists.

## Introduction

During the economic transition period, the business environment is changing rapidly. How to obtain external resources to promote corporate growth for “new and weak” start-ups has gradually become an urgent problem to be solved in academic and practical circles. The trust of venture capitalists in entrepreneurs, as one of the important ways for start-ups to obtain external “soft” resources, affects the success or failure of start-ups to a large extent. Studies have confirmed that the trust of venture capitalists in entrepreneurs helps entrepreneurs to obtain financial capital and strategic advice for new ventures, thereby enhancing the level of innovation (Maula et al., 2013) and improving financial performance (Park and Steensma, 2012). However, existing studies have paid more attention to the trust of venture capitalists in entrepreneurs after investment, and research on initial trust before investment is rare. The development of trust occurs in stages (Schoorman et al., 2007), so the investigation of trust in entrepreneurs by venture capitalists should be dynamic (Yang and Li, 2018). Initial trust is the general expectation of a party before cooperation to rely on others and the consequent behaviors based on his or her own life experience and human nature (Yi and Zhou, 2011). This trust is generated before venture capitalists make investment decisions, and it is an important factor affecting the cooperative relationship between venture capitalists and entrepreneurs (Cholakova and Clarysse, 2015). Based on this, identifying the influencing factors and the mechanism of the initial trust of venture capitalists has important theoretical and practical significance for promoting cooperation between the parties and the development of start-ups.

Initial trust, as an expectation of subjective will, is based on the non-interactive communication between parties and represents the “primary trust” of one party in the other. At present, the research on initial trust mainly focuses on two aspects. First is stage division. According to the dynamic development process of trust, trust can be divided into stages of establishment, continuation, and extinction (Rousseau et al., 1998). Among them, initial trust is not only the first step but also an important step, which is widely recognized by most scholars. For example, Yi and Zhou (2011) highlighted the initial trust stage in the evolution of trust in the study of the dimensions of trust between Chinese VC-E. Second is contributing factors. The initial trust facilitating is mainly divided into three categories: one is the characteristics of the relying party, such as trust tendencies and resource ownership (Wei and Long, 2008); the second is the characteristics of the trusted party, such as reputation and ability; and the third is institutional structural factors, such as third-party protection and risk perception (McKnight et al., 2002). In general, although some studies involve initial trust studies through case analysis, most of them are based on the dynamic evolution of trust after the partnership. Some studies emphasize that in the pre-establishment stage of a business relationship, both parties can make judgments about each other's knowledge and beliefs through mechanisms such as interactive history, information search, reputation, and stereotypes to form initial trust (Huang and Wilkinson, 2013). However, the research lacks quantitative investigation of this subject, and it is necessary to deepen the understanding of the antecedents of initial trust by more empirical analyses. The impact of entrepreneurial orientation, as an extension of the entrepreneurial spirit at the organizational level, on the initial trust of venture capitalists has not received enough attention. Entrepreneurial orientation refers to the tendency of entrepreneurs to seek new business opportunities and translate them into entrepreneurial practice through daily operations and organizational tasks, and is divided into five dimensions: perseverance, enthusiasm, risk-taking, innovation, and proactivity (Santos et al., 2020). Previous studies have confirmed that entrepreneurial orientation has a significant effect on the resource acquisition of new ventures (Moss et al., 2015), which can promote the performance of new ventures through business opportunity identification (Donbesuur et al., 2020). Based on signal theory, entrepreneurs with high entrepreneurial orientation may improve the venture capitalists' cognition of their entrepreneurial ability and thus promote initial trust by releasing signals of entrepreneurial perseverance, passion, risk-taking, innovation, and proactivity. Therefore, the orientation of entrepreneurs may be one of the important factors affecting the initial trust of venture capitalists. However, there is little existing research on the relationship between the two. Based on this, the first purpose of this study is to explore the impact of entrepreneurship orientation on the initial trust of venture capitalists.

Previous studies have mainly investigated the influence mechanism of venture capitalists' trust in entrepreneurs after investment based on signal theory and social exchange theory. Social exchange theory holds that there is a two-dimensional relationship between venture capitalists and entrepreneurs, emotional and instrumental, and a two-way relationship between social and financial resource exchanges (Huang and Knight, 2015). Venture capitalists' trust in entrepreneurs is influenced more by entrepreneurs' subjective factors, such as information signals and interpersonal signals, which will enhance the exchange of financial and non-financial resources between the two parties, thus promoting the growth of new ventures and in turn strengthening mutual trust (Von Gehlen et al., 2018). However, signal theory and social exchange theory cannot fully explain the relationship between entrepreneurship orientation and the initial trust of venture capitalists. The initial trust is generated before cooperation, and the two parties do not have a basis of “reciprocity.” However, an excessive emphasis on the subjective initiative of the entrepreneur and underestimation of the heterogeneity of the audience may lead to different understandings of the entrepreneur among different audiences (Wu et al., 2020), making the social and political skills of the entrepreneur ineffective (Fisher et al., 2017). This requires entrepreneurs to strengthen their purpose-oriented management to more effectively obtain the audience's evaluation of themselves and their start-ups (Kibler et al., 2017). The impression management strategy refers to the process by which entrepreneurs actively control their self-image or corporate image to achieve a certain value purpose (Ashforth and Gibbs, 1990), which includes acquired impression management and defensive impression management (Bolino et al., 2008). Existing studies have shown that the theory of impression management can effectively explain the issue of the legitimacy of entrepreneurs in the start-up stage of enterprises facing “new entry defects” and other constraints (Nagy et al., 2012). In addition, in an achievement context, different goal orientations have different influences on how entrepreneurs make decisions and take action (Uy et al., 2017). Individuals with different goal orientations have different cognitive patterns and behavioral responses, and there are significant differences in the tendency to adopt an active strategy or avoidance strategy (Hirst et al., 2009). For example, different motivations for enterprise innovation can improve the innovation performance of brand communities through participation in the behaviors of acquired impression management and defensive impression management (Tang et al., 2020). Therefore, the different entrepreneurial orientations of entrepreneurs should have different influences on the choice of impression management strategies. Based on this, the second problem of this study is to explore the mediating effect of the difference in entrepreneurs' impression management strategy between different entrepreneurial orientations and the initial trust of venture capitalists.

Can entrepreneurs' different impression management strategies definitely generate initial trust in venture capitalists? According to the theory of impression management, an individual's impression management behavior is not only affected by role norms but also restricted by performance venues. Previous studies have shown that the effectiveness of the impression management of entrepreneurs is limited by the cognitive attributes of the audience (Pollack and Bosse, 2014). Hypocrisy perception is defined as an individual's perception of inconsistency between the words and deeds of others (Greenbaum et al., 2015). When entrepreneurs convey different signals of entrepreneurial orientation to venture capitalists through acquired impression management strategies and defensive impression management strategies, venture capitalists make different cognitive interpretations of those signals, which may affect their initial trust judgment. In other words, when venture capitalists' perception of hypocrisy is high, the impression management strategy of entrepreneurs may reduce the initial trust cognition of venture capitalists or, on the contrary, enhance the initial trust cognition of venture capitalists. Based on this, the third issue of this research is to explore the moderating effect of venture capitalists' hypocrisy perception of entrepreneurs on different entrepreneur impression management strategies and venture capitalists' initial trust.

In summary, based on signal theory and impression management theory, this research examines the influence of five different entrepreneurial orientations on the initial trust of venture capitalists, as well as the mediating effect of entrepreneurs' impression management strategies and the moderating effect of false perception. Our research will enrich the existing research on entrepreneurial orientation and initial trust of venture capitalists. Firstly, taking five dimensions of entrepreneurial orientation as the antecedent variable of initial trust of venture capitalists, it fills the gap of the existing research on the relationship to a certain extent. Our research will provide a signal theory explanation for why entrepreneurial orientation affects the initial trust of venture capitalists. Secondly, by introducing the impression management strategies of entrepreneurs, our study will reveal how the entrepreneurs' individual entrepreneurial orientation influences the initial trust of venture capitalists through their different impression management strategies. This will provide a new theoretical perspective to explore how entrepreneurial orientation affects the initial trust process of venture capitalists. Finally, by examining the moderating effect of venture capitalists' perception of hypocrisy, we will define the boundary of effectiveness of different impression management strategies. The theoretical significance of the research is that it will help researchers clarify the relationship and mechanism between entrepreneurial orientation and initial trust, and the practical significance is that it will assist venture capitalists in correctly understanding entrepreneurial orientation, enhancing initial trust and promoting entrepreneurial cooperation.

## Theoretical Overview and Hypotheses

### Effect of the Signal of Entrepreneurial Orientation on Venture Capitalists' Initial Trust

At present, there are two main disputes about the dimension of entrepreneurial orientation in academia. One is the single dimension of entrepreneurial orientation, which includes risk-taking, innovation, and proactiveness (Covin and Slevin, 1989). They believe that entrepreneurial orientation is an organizational attribute that entrepreneurs show in organizations or business units. This view assumes that there are risk-taking, innovation, and proactiveness in entrepreneurial orientation, and emphasizes that the research of entrepreneurial orientation needs to start from the whole rather than the part (Gupta and Gupta, 2015). The other one is the multidimensional entrepreneurial orientation, which includes risk-taking, innovativeness, proactiveness, autonomy, and competitive aggressiveness (Lumpkin and Dess, 1996). They conceptualize entrepreneurial orientation as a kind of entrepreneurial spirit, and as long as there is an attribute in the five dimensions, they can use the entrepreneur label to define it. Nowadays, most researches mainly focus on the influence of entrepreneurial orientation at the enterprise level on organizational creativity, resource acquisition, and enterprise performance (Ma and Yan, 2016; Shan et al., 2016; Li Y. et al., 2018). It is limited by the measurement of entrepreneurial orientation dimension (Covin and Lumpkin, 2011; Covin and Wales, 2012); there are few studies on the effectiveness of individual entrepreneurship orientation (Koe, 2016; Rahim et al., 2018; Gao et al., 2020). Scholars have also conducted a series of explorations on the dimension measurement of individual entrepreneurship orientation, and the research has gained progress to some extent. For example, Santos et al. (2020) based on previous research added two new dimensions of entrepreneurship orientation: entrepreneurial passion and entrepreneurial perseverance. Integrating the characteristics, emotions, and behaviors of entrepreneurs, they proposed five dimensions of entrepreneurship orientation: perseverance, passion, risk-taking, innovation, and proactivity orientation.

Initial trust is the general expectation of a party to be able to rely on others and their behaviors based on that party's own life experience and understanding of human nature (Yi and Zhou, 2011). In the initial stage of establishing the relationship between venture capitalists and entrepreneurs, since there is no history of interaction between the two sides, venture capitalists cannot make judgments based on previous exchanges between the two sides, and their trust in entrepreneurs can be established only on the basis of available information about the entrepreneurs (Huang and Wilkinson, 2013). Signal theory indicates that the transmission of signals occurs mainly through the sender, signal, and receiver (Connelly et al., 2011). On the basis of signals sent by entrepreneurs to venture capitalists, the venture capitalists may judge the entrepreneurs' abilities and behaviors according to the signal content and then decide whether to trust them. Therefore, the signal of different start-up orientations of entrepreneurs may have a positive impact on the initial trust of venture capitalists.

First, perseverance is the most obvious entrepreneurial trait. Perseverance is regarded as one of the most important characteristics of successful entrepreneurs (Shane et al., 2003). Entrepreneurs can succeed in starting their own businesses only if they persist in pursuing their goals. Perseverance orientation refers to the entrepreneurial trait of persistence in pursuing goals even if entrepreneurial obstacles are encountered (Gerschewski et al., 2016). In the early stages of the establishment or development of start-ups, facing the development dilemma of “newcomers' weakness,” entrepreneurs who persist in entrepreneurship are driven by entrepreneurial self-efficacy (Li C. et al., 2018), continue to promote entrepreneurial self-learning, and are willing to put more effort into sending positive signals to venture capitalists, thereby enhancing the venture capitalists' positive expectations. When making initial trust judgments, venture capitalists will consider not only the return if the venture project succeeds but also the entrepreneur's ability to complete the project. Entrepreneurs who persist in entrepreneurship orientation have high psychological resilience (Eisenberger and Leonard, 1980). In the face of entrepreneurial pressure and difficulties, they are better able to actively resist adversity and quickly adapt to changes. This signals to VC the potential of entrepreneurs to recover and succeed in difficult situations. At the same time, strong motivation for goal realization can better meet the expectations of venture capitalists for the results of venture cooperation.

Second, passion is the most direct entrepreneurial experience for entrepreneurs. Passionate orientation means that entrepreneurs consciously acquire and experience positive and strong emotion in the entrepreneurial process (Cardon et al., 2009). In the early stage of social interaction, passionate entrepreneurs actively participate in the establishment of start-ups, showing positive emotions of self-confidence and optimism, which easily release their emotions, attitudes, and behavior signals. Such signals are likely to infect venture capitalists and promote the rapid establishment of a trust relationship between the two parties. Individuals who are passionate about entrepreneurship have a strong sense of identity, which drives them to continuously acquire new knowledge and skills to achieve entrepreneurial success (Zhang and Li, 2019). This signals to venture capitalists that entrepreneurs are coachable. Ciuchta et al. (2018) confirmed that the coachability of entrepreneurs is a kind of interpersonal signal, and the higher the perception of entrepreneurs' coachability is, the more willing venture capitalists are to invest. Emotion has a transmitting function. The positive emotional experience of venture capitalists comes not only from the entrepreneur's passion for starting a business but also from the passion for perceiving products or services (Davis et al., 2017). This dual emotional experience may enhance the strength of the signal. Research has shown that when individuals experience positive emotional states, they are more likely to establish cooperation (Dimotakis et al., 2012).

Third, risk-taking reflects the entrepreneur's tendency to take risks. This entrepreneurial orientation refers to the tendency to take chances when facing an uncertain environment, dare to venture into unknown areas, and be willing to take risks (Langkamp Bolton and Lane, 2012). Alvarez and Busenitz (2001) pointed out that entrepreneurial companies tend to take risks in turning new products and services into market opportunities to create new wealth. In the process of developing new products and new services, facing high market uncertainty and investment return risks, entrepreneurs' strong risk responsibility enables startups to identify potential risks that may exist and quickly develop market response strategies to reduce risks in order to promote enterprise development, which releases positive signals of entrepreneurial competence to venture capitalists. Entrepreneurs may also use rhetoric to describe their image of daring to take risks, and to enhance the signal strength of the initial trust of venture capitalists. Wang et al. (2016) pointed out in their study that different language styles of project sponsors can change investors' perception of project prospects and thus affect their investment intentions.

Fourth, innovation reflects the entrepreneur's tendency to pursue excellence. Innovation orientation refers to the creative and experimental behavior tendency to develop new products, new services, and new technologies through new processes (Langkamp Bolton and Lane, 2012). In the early stage of project funding screening, venture capitalists pay more attention to the products or opportunities provided by entrepreneurs than the quality of the entrepreneurs (Mitteness et al., 2012). However, at this stage, venture capital is usually based on a single product, a few products, or even products that have not been fully formed or tested (Parhankangas and Ehrlich, 2014), leading to a lack of objective evidence for the success of the product or service market for the venture capitalists, who then can make investment decisions only through subjective judgment (Maxwell et al., 2011). Entrepreneurs with an innovative entrepreneurial orientation have a stronger innovation drive and higher demand for entrepreneurial resources. They usually seek external support for product or service development or process reengineering, and products or services are the focus of entrepreneurship speech (Mollick, 2014). Creative products or services can gain widespread recognition and competitive advantages (Ward, 2004), which undoubtedly strengthens the signal to venture capitalists to release high-quality products or services.

Last, proactivity reflects the tendency of entrepreneurs to venture to be a pioneer. Proactivity orientation refers to the tendency to lead competitors and meet future demands by discovering and seizing opportunities, and introducing new products and services (Langkamp Bolton and Lane, 2012). Taking the initiative and taking the lead in participating in emerging markets is the cornerstone of entrepreneurs' behaviors (Qin et al., 2017). Entrepreneurs with a strong proactivity orientation are unwilling to maintain the existing status quo, seek opportunities for transformation, and promote updates of products or services by introducing new technologies or business models, thus becoming “leaders” of industry. Hu et al. (2018) confirmed that the proactive personalities of individuals are positively related to their entrepreneurial alertness, which in turn influences entrepreneurial intention. This proactive entrepreneurial tendency helps signal to venture capitalists that entrepreneurs have more potential to innovate and bring profit. Research by Anglin et al. (2018) confirmed that project sponsors who have a positive attitude toward “taking necessary measures to achieve established goals” are more likely to succeed in obtaining financing. Entrepreneurs with a strong proactivity orientation are market founders rather than followers who actively carry out innovation (Moss et al., 2015). Compared with other entrepreneurs, it has more acquired competitive advantages, which can transmit high-quality signals to venture capitalists, and help to enhance their perception of entrepreneurial ability (He et al., 2020). Given the above analysis, we propose the following hypothesis:

Hypothesis 1a (H1a): The signal of entrepreneurs' perseverance orientation is positively related to venture capitalists' initial trust.

Hypothesis 1b (H1b): The signal of entrepreneurs' passion orientation is positively related to venture capitalists' initial trust.

Hypothesis 1c (H1c): The signal of entrepreneurs' risk-taking orientation is positively related to venture capitalists' initial trust.

Hypothesis 1d (H1d): The signal of entrepreneurs' innovation orientation is positively related to venture capitalists' initial trust.

Hypothesis 1e (H1e): The signal of entrepreneurs' proactivity orientation is positively related to venture capitalists' initial trust.

### The Mediating Role of Impression Management Strategies

According to the content of the signal, it can be divided into quality signals and intention signals. Quality signals convert invisible capabilities into externally observable signals, while intention signals indicate the direction of organizational behavior. That is, in addition to different entrepreneurial orientations that have a direct impact on the initial trust of venture capitalists, entrepreneurs can also intentionally release signals to the outside world to influence venture capitalists' perceptions of their abilities and judgment, so that they can then consider whether to give trust. How do entrepreneurs release signals through which their entrepreneurial orientation can affect the initial trust of venture capitalists? From the perspective of social psychology, individuals not only pay close attention to how others view and evaluate them but also change their behavior based on others' views (Leary and Kowalski, 1990). Entrepreneurs' impression management strategies refer to a kind of means and manifestation that entrepreneurs use to influence others' perception of their individual or corporate image (Eisenberger and Leonard, 1980; Elsbach and Sutton, 1992), including acquired impression management strategies and defensive impression management strategies (Bolino et al., 2008). Acquired impression management strategies are strategies to improve others' positive perceptions and to conceal one's negative image. Different goal-oriented individuals have different cognitive and behavioral responses, and there are significant differences in whether they adopt proactive or avoidant strategies (Hirst et al., 2009). Therefore, in the process of releasing signals, entrepreneurs with different entrepreneurial orientations may adopt different impression management strategies to transmit signals, which in turn affects the initial trust of venture capitalists.

With high pressure, multiple obstacles, and high risk in the process of entrepreneurship, entrepreneurs who persist in the process often defy difficulties, constantly strengthen self-learning, and cope with a complex and changing external environment. This perseverance can be positively recognized by venture capitalists. In addition, in the face of business setbacks and failures, persistent entrepreneurs actively adjust their mentality and strive to overcome difficulties and meet challenges with an optimistic attitude. This kind of strong cognitive resilience is likely to be praised by venture capitalists. In addition, entrepreneurs' resilience cannot be separated from social support (Zhang and Li, 2020). In other words, entrepreneurs who have received social support tend to care more about others' positive evaluations of them and thus have more confidence in their own entrepreneurial capabilities (Zhang and Li, 2019). Therefore, to obtain the social support and initial trust of venture capitalists, entrepreneurs who have a high perseverance orientation may release perseverance signals by acquired impression management strategies. For example, entrepreneurs may demonstrate their perseverance and cognitive resilience to venture capitalists through self-improvement or adopt strategies such as actively taking responsibility after setbacks and failures so that they will be recognized and trusted on the basis of their “good impression.”

In the face of an uncertain environment, entrepreneurs with a passion orientation usually have a high-risk tolerance. To adapt to rapid changes in development, they dare to make attempts and to make mistakes. Driven by self-identity and positive emotion, they will actively carry out entrepreneurial learning to improve their self-cognitive flexibility so that they can use existing resources to flexibly respond to entrepreneurial challenges. Studies have shown that a learning goal orientation can improve entrepreneurs' self-efficacy (Liu et al., 2019) and promote effective reasoning (Deng et al., 2020). The active efforts of passionate entrepreneurs in this process may be recognized and affirmed by venture capitalists. In addition, the entrepreneurial passion of entrepreneurs can affect stakeholders through emotional contagion. For example, researchers have found that venture capitalists prefer to invest in passionate entrepreneurs (Chen et al., 2009). Therefore, to gain the initial trust of venture capitalists in such a start-up situation, entrepreneurs with a passion orientation tend to arouse their active psychological state and strive to present a good impression to venture capitalists. Studies have shown that passionate entrepreneurs tend to seek external financial support and maintain long-term development with stakeholders according to different decision-making styles and strategic types (Cardon et al., 2013). Therefore, entrepreneurs who are passionate about entrepreneurship may adopt acquired impression management strategies, such as demonstration and using positive language to release passion signals.

Risk-taking entrepreneurs usually have a high degree of risk tolerance. They invest their resources in high-risk projects or technical fields in the market. Once the resources are marketed, they will obtain a higher return on investment. If not, their corporate performance will be greatly reduced (Si et al., 2016). Although the entrepreneurial orientation of risk-taking can release positive and optimistic signals of risk-taking, if entrepreneurs overemphasize their risk-taking when they interact with venture capitalists, the venture capitalists may doubt their optimistic attitude of “blind” self-confidence and worry about their ability to resist risks. To address such concerns among venture capitalists, entrepreneurs may be more careful in weighing the advantages and credibility issues of such signaling through impression management strategies. For example, excessive emphasis on the benefits of positive impression management in releasing risk-taking signals may be counterproductive. Therefore, to weaken the potential threats associated with risk-taking signals, compared with acquired impression management strategies, entrepreneurs may release signals by adopting defensive impression management strategies.

Innovation-oriented entrepreneurs are driven mainly by innovation. They emphasize the reengineering of products or services, break the existing market balance, and provide customers with creative products or services. Although this approach can release positive signals of innovative potential, it may also give venture capitalists a negative impression. This negative impression comes from two main aspects: on the one hand, entrepreneurs pay too much attention to products or services and then ignore market and customer information, which leads to misjudgment of future products and undermines their start-ups (Wales et al., 2013). On the other hand, venture capitalists and market customers have different perceptions of the legitimacy of the entrepreneurs. Investors pay more attention to the prospects of business development, while entrepreneurs pay more attention to the product itself (Aldrich and Fiol, 1994). In addition, to cope with the threat of competitor imitation, avoid peer competition, and reduce external supervision, entrepreneurs may avoid communicating with the audience to prevent the spread of information (Kibler et al., 2017). Therefore, entrepreneurs may adopt defensive impression management strategies to release signals. On the one hand, they may enhance the good impression of their business in the eyes of venture capitalists, and on the other hand, they may also avoid external pressure and threats.

Proactivity-oriented entrepreneurs are not satisfied with the current situation, actively seek change, constantly try new approaches, and invest much time and energy in exploring new market opportunities. Although this can signal the potential of entrepreneurs to become market leaders, it may also give venture capitalists a negative impression. In the view of venture capitalists, such entrepreneurs may spend much time and energy exploring market opportunities, which may scatter their energy and cause a lack of a unique focus, resulting in insufficient utilization of enterprise resources and causing venture capitalists to worry about the entrepreneurial ability to take advantage of opportunities (Li Y. et al., 2018). In other words, taking the lead in demonstrating proactive and other acquired impression management to shape themselves or improve the impression they make on venture capitalists may have counterproductive effects. Therefore, to reduce the negative impact of proactivity signals on self-image, entrepreneurs may adopt defensive impression management in releasing signals. Research has shown that in emerging market, to avoid potential negative results, entrepreneurs usually adopt defensive impression management strategies, such as public statements, demarcations, and hedging, to label the market (Granqvist et al., 2013). Based on the above discussion, we propose the following hypothesis:

Hypothesis 2a (H2a): The acquired impression management strategy plays a mediating role between entrepreneurs' perseverance orientation and venture capitalists' initial trust.

Hypothesis 2b (H2b): The acquired impression management strategy plays a mediating role between entrepreneurs' passion orientation and venture capitalists' initial trust.

Hypothesis 2c (H2c): The defensive impression management strategy plays a mediating role between entrepreneurs' risk-taking orientation and venture capitalists' initial trust.

Hypothesis 2d (H2d): The defensive impression management strategy plays a mediating role between entrepreneurs' innovation orientation and venture capitalists' initial trust.

Hypothesis 2e (H2e): The defensive impression management strategy plays a mediating role between entrepreneurs' proactivity orientation and venture capitalists' initial trust.

### The Moderating Role of Perceptions of Hypocrisy

Perceptions of hypocrisy refer to the individual's perception of the inconsistency between the words and deeds of others (Greenbaum et al., 2015). Perceptions of hypocrisy emphasize individuals' subjective feelings toward others' behavior, which may lead to the judgment that others are hypocritical, regardless of whether they engage in unreasonable or unethical behavior (Yao et al., 2019). At present, most scholars have studied the hypocrisy of leadership in the field of organizational behavior and corporate hypocrisy in the field of marketing, but less research has been conducted on the perception of hypocrisy in the field of entrepreneurship. However, some studies point out that the consistency of entrepreneurs' behaviors is an important criterion for venture capitalists to evaluate entrepreneurs' credibility (Chen and Ye, 2010) and is positively correlated with venture capitalists' investment decisions (Yang and Li, 2018).

The impression management strategies reflect entrepreneurs' intentional “self-presentation” in order to gain initial trust. We believe that there is still a gap between the entrepreneurial impression management strategy and the initial trust of venture capitalists. In other words, in the process of signal transmission, whether entrepreneurs' different impression management strategies can be transformed into initial trust depends on how venture capitalists interpret them. Previous studies have pointed out that the effectiveness of impression management strategies depends largely on the different perceptions of audiences (Kibler et al., 2017). As subjective perceptions, venture capitalists' perceptions of entrepreneurs' differences in words and deeds vary. The perception of hypocrisy means that venture capitalists make negative judgments about the consistency of entrepreneurial behavior, which will increase the potential risk to the initial trust of venture capitalists. Therefore, the perception of hypocrisy may weaken the relationship between entrepreneurs' impression management strategy and the initial trust of venture capitalists. Specifically, the higher the perception of entrepreneurs' hypocrisy, the more malicious intent will be attributed to the entrepreneurs' behavioral strategy. Especially when entrepreneurs use the defensive impression management strategy to release signals of daring to take risks, pursuing product or service innovation, and actively seeking market opportunities, this minimizing positive effect may enhance venture capitalists' perception of the inconsistency of words and deeds of entrepreneurs. In the view of venture capitalists, these perceptions are only a superficial illusion, but the real purpose is to pursue profit, which will lead them to question the behavioral motivation of entrepreneurs and trigger their resistance. Studies have shown that when individuals have a high perception of other people's hypocrisy, it is likely to cause negative interpersonal reactions, such as distrust and moral condemnation (Effron et al., 2018). In contrast, the lower the perceptions of entrepreneurs' hypocrisy are, the greater the initial trust in the entrepreneurs because venture capitalists will interpret their behavioral strategies in good faith. Especially when entrepreneurs adopt the acquired impression management strategy to release signals of passion and perseverance entrepreneurial orientations, this maximum positive effect may strengthen the venture capitalists' perception of the consistency of the entrepreneurs' words and deeds and enable the entrepreneurs to consciously release different behavioral signals to better meet the trust cognition of venture capitalists on behavior consistency in entrepreneurial cooperation (Yang and Li, 2017). In summary, we propose the following hypothesis:

Hypothesis 3a (H3a): Perceptions of hypocrisy negatively moderate the relationship between the acquired impression management strategy and venture capitalists' initial trust.

Hypothesis 3a (H3a): Perceptions of hypocrisy negatively moderate the relationship between the defensive impression management strategy and venture capitalists' initial trust.

**Figure 1:**
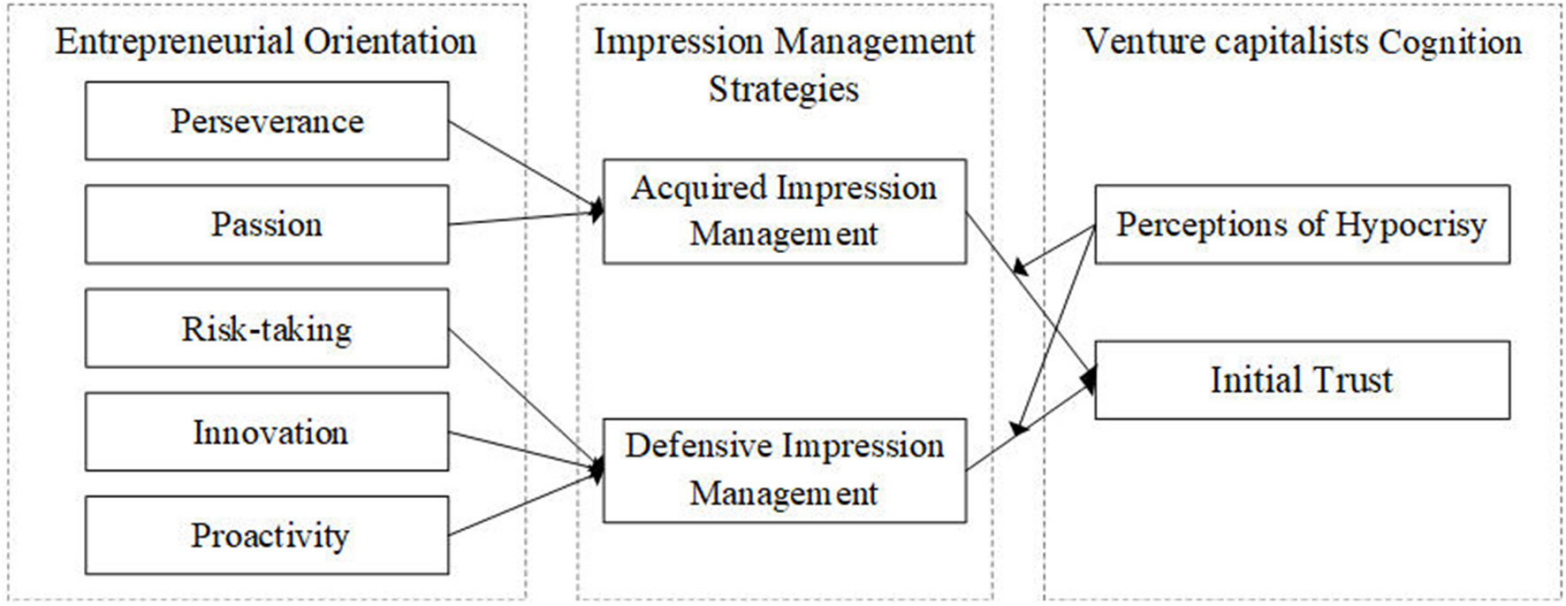
Theoretical model.

Based on the above theoretical view and research hypotheses, the theoretical model of this research is shown in Figure 1.

## Methods

### Participants and Procedures

Taking into account the research on venture capitalists' initial trust in entrepreneurs before the cooperative relationship, according to the definition of new ventures by McDougall and Robinson (1990), venture capitalists whose investment company was established within the previous 8 years are selected as the research objects. We adopted the form of electronic questionnaire and commissioned a professional market research company to conduct online data collection for venture capitalists in various regions of the country. Before the research started, the research team did a lot of preparatory work. In terms of questionnaire design, in order to ensure the accuracy and authenticity of the online survey, the research team developed the questionnaire by combing relevant literature and conducting field research. The team members communicated and discussed repeatedly to improve the questionnaire design. During the release of the questionnaire, team members communicated with the research company many times to inform and supervise the quality of the questionnaire survey. For example, the survey respondents were informed that venture capitalists must have experience in investing in startup entrepreneurs. Finally, the collected questionnaires were screened to eliminate invalid questionnaires, such as those with short answer time and incomplete answers. The data collection period was from July 2020 to September 2020. A total of 357 questionnaires were collected, of which 202 were valid. The questionnaire recovery rate was 56.58%. The descriptive statistical results of the sample are as follows: Among the venture capitalists surveyed, there were 107 male venture capitalists, accounting for 53%, and 95 female venture capitalists, accounting for 47%. In terms of age, 28 were aged 25 and below, accounting for 13.9%; 120 were aged 26–35, accounting for 59.4%; 42 were aged 36–45, accounting for 20.8%; and 12 were aged 46 and above, accounting for 5.9%. In terms of education level, 31 had completed high school or below, accounting for 15.3%; 70 had a college degree, accounting for 34.7%; 89 had an undergraduate education, accounting for 44.1%; and 12 had a postgraduate degree and above, accounting for 5.9%. In terms of entrepreneurial investment experience, 63 had entrepreneurial experience, accounting for 31.2%, and 139 had no previous entrepreneurial experience, accounting for 68.8%. Judging by the establishment years of the invested companies, 149 venture capitalists had invested in companies for 1–3 years, accounting for 73.8%; 48 had invested for 4–6 years, accounting for 23.8%; and 5 had invested for 8 years, accounting for 2.5%.

### Measures

The measurement scales used in this study are all scales with good reliability and validity in the Chinese and English literature, and they were ultimately determined after back-translation procedures. Except for controlled variables, all variables were scored with a five-point Likert scale ranging from 1 (totally disagree) to 7 (totally agree). The measurement of the independent variable entrepreneurial orientation was based on the scale developed by Santos et al. (2020) with five dimensions, perseverance orientation, passion orientation, risk-taking orientation, innovation orientation, and proactivity orientation, with a total of 12 items. Perseverance had three items; an example is “In complex situations, even if others choose to give up, entrepreneurs still insist on achieving their goals,” and the Cronbach's α was 0.89. Passion had two items; an example is “Entrepreneurs have a passion for finding good business opportunities, developing new products or services, exploiting business applications and creating new solutions for existing problems and needs,” and the Cronbach's α was 0.85. Risk-taking had two items; an example is “Entrepreneurs like to venture into the unknown and make risky decisions,” and the Cronbach's α was 0.77. Innovation had three items; an example is “Entrepreneurs favor trying out new approaches to problem solving rather than using methods that others often use,” and the Cronbach's α was 0.89. Proactivity had two items; an example is “Entrepreneur tend to plan projects in advance,” and the Cronbach's α was 0.84. The scale in the study of Zhao and Zhao (2019), which had 15 items, was used to measure the mediator variable impression management strategies, including two dimensions: acquired impression management strategies and defensive impression management strategies. Acquired impression management strategies had seven items, an example is “In order to make a good impression on me, entrepreneurs always actively participate in start-up discussions with me during the start-up process,” and the Cronbach's α was 0.87. Defensive impression management strategies had eight items; an example is “In order to avoid losing face when discussing with me the idea of starting a business project, entrepreneurs often agree with my opinion instead of arguing with me,” and the Cronbach's α was 0.91. The moderator variable perceptions of hypocrisy were measured with a four-item scale developed by Greenbaum et al. (2015); an example is “The entrepreneur asked me to follow the rules, but he/she violated them,” and the Cronbach's α was 0.92. The dependent variable initial trust was measured using a scale from Yi (2011) that contained four items; an example is “I think entrepreneurs are capable and persist in completing tasks,” and the Cronbach's α was 0.89. Additional demographic characteristic variables, including gender, age, education level, entrepreneurial experience of entrepreneurs, and the established years of invested enterprises, were selected as control variables.

## Results

### Common Method Bias Analyses

The sample data were collected by questionnaire in our study, and the measurement items were answered by the venture capitalists themselves, which may incur the problem of common method bias. To ensure the reliability of the research results, we used SPSS 21.0 for Harman's single-factor test and carried out principal component analysis (PCA) on all measurement items of the core variables involved in the research. The data results show that among the seven principal components extracted by the unrotated component matrix, there is no single component that can explain the overall variation, and the first principal component with the largest eigenvalue explains the variation of 26.48%, less than half the overall variation of 70.22%, which shows that there is no serious common method bias in our study.

### Confirmatory Factor Analyses

We conducted confirmatory factory analyses (CFAs) to test the discriminant validity of the research variables included in our study: perseverance orientation, passion orientation, risk-taking orientation, innovation orientation, proactivity orientation, acquired impression management strategies, defensive impression management strategies, perceptions of hypocrisy, and initial trust. As shown in Table 1, our hypothesized nine-factor model fit the data better [χ2/df = 1.627, CFI = 0.928, TLI = 0.919, RMSEA = 0.056] than the other factor models such as the eight-factor and seven-factor models. These results provided support for the distinctiveness of the nine variables.

**Table 1:** Confirmatory factory analyses.

**Model**	**χ^2^**	**df**	**χ^2^/df**	**Δχ2(Δdf)**	**CFI**	**TLI**	**RMSEA**
Nine-factor model	852.63	524	1.63		0.93	0.92	0.06
Eight-factor model^a^	948.22	532	1.78	95.60^***^(8)	0.91	0.90	0.06
Seven-factor model^b^	1179.90	539	2.19	327.27^***^(15)	0.86	0.85	0.08
Six-factor model^c^	1271.66	545	2.33	419.03^***^(21)	0.84	0.83	0.08
Five-factor model^d^	1503.32	550	2.73	650.69^***^(26)	0.79	0.78	0.09
Four-factor model^e^	2212.15	554	3.99	1359.53^***^(30)	0.64	0.61	0.12
Three-factor model^f^	2809.14	557	5.04	1956.51^***^(33)	0.51	0.48	0.14
Two-factor model^g^	3457.91	559	6.19	2605.28^***^(35)	0.37	0.33	0.16
One-factor model	3544.29	560	6.33	2691.66^***^(36)	0.35	0.31	0.16

*N = 202. PE, perseverance orientation; PA, passion orientation; RT, risk-taking orientation; IN, innovation orientation; PR, proactivity orientation; AIM, acquired impression management strategies; DIM, defensive impression management strategies; PH, perceptions of hypocrisy; IT, initial trust*.

a *In the eight-factor model, items of PE and PA were loaded on one factor*.

b *In the seven-factor model, items of RT, IN, and PR were loaded on one factor*.

c *In the six-factor model, items of PE and PA were loaded on one factor, and items of RT, IN, and PR were loaded on one factor*.

d *In the five-factor model, items of PE, PA, RT, IN, and PR were loaded on one factor*.

e *In the four-factor model, items of PE, PA, RT, IN, and PR were loaded on one factor, and items of AIM and DIM were loaded on one factor*.

f *In the three-factor model, items of PE, PA, RT, IN, and PR were loaded on one factor, items of AIM, DIM and PH were loaded on one factor*.

g *In the two-factor model, items of PE, PA, RT, IN, PR, IM, DIM, and PH were loaded on one factor*.

*** *p < 0.001*.

### Preliminary Analyses

Table 2 provides the mean, standard deviation, and correlations among all variables. Table 2 shows that perseverance orientation (r = 0.461, p < 0.01), passion orientation (r = 0.511, p < 0.01), risk-taking orientation (r = 0.506, p < 0.01), innovation orientation (r = 0.416, p < 0.01), and proactivity orientation (r = 0.351, p < 0.01) are significantly and positively correlated with initial trust. H1a, H1b, H1c, H1d, and H1e are therefore preliminarily supported. Moreover, acquired impression management strategies are obviously positively correlated with perseverance orientation (r = 0.317, p < 0.01), passion orientation (r = 0.302, p < 0.01), and initial trust (r = 0.269, p < 0.01); H2a and H2b are therefore preliminarily supported. Defensive impression management strategies are positively related to risk-taking orientation (r = 0.300, p < 0.01), innovation orientation (r = 0.252, p < 0.01), proactivity orientation (r = 0.382, p < 0.01), and initial trust (r = 0.526, p < 0.01), H2c, H2d, and H2e are therefore preliminarily supported. There is a negative correlation between perceptions of hypocrisy and acquired impression management strategies (r = −0.167, p < 0.05), which indicates that perceptions of hypocrisy may have a negative effect on acquired impression management strategies.

**Table 2:** Means, standard deviations, and correlations for all variables.

**Variables**	**1**	**2**	**3**	**4**	**5**	**6**	**7**	**8**	**9**	**10**	**11**	**12**	**13**	**14**
1.Gender	-													
2.Age	−0.14	-												
3.Education	−0.15^*^	0.27^**^	-											
4.Experience	−0.05	0.12	0.10	-										
5.Establishment	−0.13	0.21^**^	0.15^*^	−0.02	-									
6.PE	−0.06	0.28^**^	0.37^**^	0.15^*^	0.16^*^	-								
7.PA	−0.04	0.36^**^	0.43^**^	0.13	0.17^*^	0.62^**^	-							
8.RT	0.13	0.29^**^	0.35^**^	0.07	0.02	0.42^**^	0.52^**^	-						
9.IN	−0.07	0.32^**^	0.28^**^	0.16^*^	0.07	0.28^**^	0.34^**^	0.34^**^	-					
10.PR	−0.03	0.32^**^	0.33^**^	0.08	0.12	0.35^**^	0.39^**^	0.51^**^	0.35^**^	-				
11.AIM	−0.02	0.10	0.12	0.03	−0.00	0.32^**^	0.30^**^	0.12	0.13	0.03	-			
12.DIM	0.14^*^	0.09	0.13	0.08	−0.01	0.09	0.13	0.30^**^	0.25^**^	0.38^**^	0.01	-		
13.PH	0.08	−0.03	−0.11	0.06	−0.15^*^	−0.13	−0.13	−0.22^**^	−0.19^**^	−0.23^**^	−0.17^*^	0.01	-	
14.IT	0.04	0.27^**^	0.32^**^	0.14	0.04	0.46^**^	0.51^**^	0.56^**^	0.42^**^	0.35^**^	0.27^**^	0.53^**^	−0.13	-
Mean	0.53	2.19	2.41	0.31	1.29	3.911	4.00	3.92	3.96	3.91	2.70	3.99	2.35	4.13
SD	0.50	0.74	0.82	0.46	0.51	1.03	1.05	1.00	1.07	1.08	1.08	0.83	1.23	0.91

* *p < 0.05*,

** *p < 0.01*.

### Analyses of the Main Effect and Mediating Effect

According to the steps proposed by MacKinnon (2008), we used the SPSS PROCESS program developed by Hayes (2013) and referred to model 4 (using the bootstrap method to run 5,000 iterations) in the process macro plug-ins, examining whether the acquired impression management strategies mediate the relationship between perseverance orientation, passion orientation, and the initial trust of venture capitalists and whether defensive impression management strategies mediate the relationship between risk-taking orientation, innovation orientation, proactivity orientation, and the initial trust of venture capitalists.

Regarding the mediating effect of acquired impression management strategies, the specific results are shown in Table 3. After controlling for gender, age, education level, experience, and enterprise establishment years, the first step is to test the total effect of perseverance orientation, passion orientation, and the initial trust of venture capitalists. Model M1 and M2 in Table 3 show that perseverance orientation (M1, β = *0.324, p* < *0.001*) and passion orientation (M2, β = *0.371, p* < *0.001*) have significant positive effects on the initial trust of venture capitalists. Therefore, H1a and H1b are further supported. The second step is to examine the direct influence of perseverance orientation and passion orientation on acquired impression management strategies. M3 and M4 in Table 3 show that perseverance orientation has a significant positive impact on acquired impression management strategies (M3, β = *0.340, p* < *0.001*). Passion orientation has a significant positive impact on acquired impression management strategies (M4, β = *0.330, p* < *0.001*). The third step is to examine the relationship between the acquired impression management strategies and the initial trust of venture capitalists. M5 and M6 indicate that when perseverance orientation and passion orientation are controlled respectively, there is a significant regression relationship between acquired impression management strategies and the initial trust of venture capitalists (M5, β = *0.113, p* < *0.05*; M6, β = *0.108, p* < *0.05*). In addition, the bootstrap method based on the percentile deviations shows that the mediating effect between perseverance orientation and the initial trust of venture capitalists is significant (β = *0.038, SE* = *0.021*, 95% confidence interval [0.006, 0.086]), and the mediating effect accounts for 11.8% of the total effect; in addition, the mediating effect between passion orientation and the initial trust of venture capitalists is significant (β = *0.036, SE* = *0.020*, 95% confidence interval [0.001, 0.079]), and the mediating effect accounts for 9.6% of the total effect. Therefore, H2a and H2b are supported.

**Table 3:** The mediation effect test of acquired impression management strategies.

**Variable**	**IT**		**AIM**		**IT**	
	**M1**	**M2**	**M3**	**M4**	**M5**	**M6**
Gender	0.19	0.16	−0.02	−0.05	0.19	0.16
Age	0.17^*^	0.12	0.03	−0.01	0.17^*^	0.12
Education	0.18^*^	0.14	0.00	−0.02	0.18^*^	0.14
Experience	0.11	0.12	−0.05	−0.032	0.11	0.12
Establishment	−0.11	−0.11	−0.12	−0.12	−0.09	−0.10
PE	0.32^***^		0.34^***^		0.29^***^	
PA		0.37^***^		0.33^***^		0.34^***^
AIM					0.11^*^	0.11^*^
Intercept	2.05^***^	2.07^***^	1.49^***^	1.62^***^	1.89^***^	1.90^***^
*R*^2^	0.27	0.29	0.10	0.10	0.29	0.31
F	11.94^***^	13.56^***^	3.78^**^	3.39^**^	11.04^***^	12.42^***^

*Reported coefficients are unstandardized (with robust standard errors)*.

* *p < 0.05*,

** *p < 0.01*,

*** *p < 0.001*.

Regarding the mediating effect of defensive impression management strategies, the analysis results are shown in Table 4. After controlling for gender, age, education level, experience, and enterprise establishment years, the first step is to test the total effect of risk-taking orientation, innovation orientation, proactivity orientation, and the initial trust of venture capitalists. M7, M8, and M9 in Table 4 show that risk-taking orientation (M7, β = *0.442, p* < *0.001*), innovation orientation (M8, β = *0.272, p* < *0.001*), and proactivity orientation (M9, β = *0.380, p* < *0.001*) have significant positive effects on the initial trust of venture capitalists. Therefore, H1c, H1d, and H1e are further supported. The second step is to examine the direct influence of risk-taking orientation, innovation orientation, and proactivity orientation on acquired impression management strategies. M10, M11, and M12 in Table 4 show that risk-taking orientation (M10, β = *0.217, p* < *0.001*), innovation orientation (M11, β = *0.179, p* < *0.001*), and proactivity orientation (M12, β = *0.295, p* < *0.001*) have a significant positive impact on defensive impression management strategies. The third step is to examine the relationship between defensive impression management strategies and the initial trust of venture capitalists. M13, M14, and M15 indicate that when risk-taking orientation, innovation orientation, and proactivity orientation are controlled, there is a significant regression relationship between defensive impression management strategies and the initial trust of venture capitalists (M13, β = *0.426, p* < *0.05*; M14, β = *0.467, p* < *0.05*; M15, β = *0.404, p* < *0.05*). In addition, the bootstrap method based on the percentile deviations shows that the mediating effect between risk-taking orientation and the initial trust of venture capitalists is significant (β = *0.092, SE* = *0.044*, 95% confidence interval [0.021, 0.191]), and the mediating effect accounts for 20.9% of the total effect; the mediating effect between innovation orientation and the initial trust of venture capitalists is significant (β = *0.083, SE* = *0.042*, 95% confidence interval [0.126, 0.174]), and the mediating effect accounts for 30.7% of the total effect; and the mediating effect between proactivity orientation and the initial trust of venture capitalists is significant (β = *0.119, SE* = *0.043*, 95% confidence interval [0.041, 0.209]), and the mediating effect accounts for 31.3% of the total effect. Therefore, H2c, H2d, and H2e are further supported.

**Table 4:** The mediation effect test of defensive impression management strategies.

**Variable**	**IT**			**DIM**			**IT**		
	**M7**	**M8**	**M9**	**M10**	**M11**	**M12**	**M13**	**M14**	**M15**
Gender	0.02	0.21	0.17	0.20	0.29^*^	0.26^*^	−0.06	0.07^*^	0.06
Age	0.11	0.15	0.12	0.13	0.19	−0.02	0.10	0.14	0.13
Education	0.13	0.23^**^	0.17^*^	0.05	0.09	0.03	0.11	0.19^**^	0.16^*^
Experience	0.16	0.11	0.15	0.12	0.08	0.11	0.11	0.07	0.11
Establishment	−0.05	−0.07	−0.08	−0.00	−0.02	−0.05	−0.00	−0.04	−0.06
RT	0.44^***^			0.22^***^			0.35^***^		
IN		0.27^***^			0.180^**^			0.19^***^	
PR			0.38^***^			0.30^***^			0.26^***^
DIM							0.43^***^	0.47^***^	0.40^***^
Intercept	1.80^***^	2.07^***^	1.95^***^	2.86^***^	2.88^***^	2.71^***^	0.58	0.73^*^	0.86^**^
*R*^2^	0.34	0.25	0.33	0.11	0.10	0.18	0.48	0.41	0.44
F	17.08^***^	10.54^***^	15.76^***^	3.91^***^	3.55^**^	6.88^***^	25.63^***^	19.27^***^	21.76^***^

*Reported coefficients are unstandardized (with robust standard errors)*.

* *p < 0.05*,

** *p < 0.01*,

*** *p < 0.001*.

### Analyses of the Moderating Effect

This study refers to model 4 in the SPSS PROCESS program developed by Hayes (2013) to test whether perceptions of hypocrisy moderate the relationship between acquired impression management and initial trust of venture capitalists and between defensive impression management and the initial trust of venture capitalists. The analysis results are shown in Table 5. M16 shows that there is a main effect between acquired impression management strategies and the initial trust of venture capitalists (M16, β = *0.182, p* < *0.001*), and this main effect is moderated by the perception of hypocrisy (M16, β = −*0.088, p* < *0.05*), indicating that the perception of hypocrisy negatively moderates the relationship between acquired impression management and the initial trust of venture capitalists. Similarly, M17 shows that there is a main effect between defensive impression management strategies and the initial trust of venture capitalists (M17, β = *0.530, p* < *0.001*), and this main effect is moderated by the perception of hypocrisy (M17, β = −*0.100, p* < *0.05*), indicating that the perception of hypocrisy negatively moderates the relationship between defensive impression management and the initial trust of venture capitalists. Therefore, H3a and H3b are supported.

**Table 5:** The moderating effect test of perceptions of hypocrisy.

**Variable**	**M16 (IT)**			**M17 (IT)**		
	***β***	***SE***	***t***	***β***	***SE***	***t***
Gender	0.22	0.12	1.91	0.08	0.11	0.76
Age	0.23	0.085	2.80^**^	0.22	0.07	3.08^**^
Education	0.26	0.26	0.07^***^	0.22	0.07	3.15^***^
Experience	0.190	0.13	1.51	0.14	0.11	1.28
Establishment	−0.03	0.12	−0.27	−0.08	0.10	−0.78
AIM	0.18	0.05	3.35^***^			
DIM				0.53	0.06	8.48^***^
PH	−0.07	0.05	−1.41	−0.11	0.04	−2.55^*^
AIM × PH	−0.09	0.04	−2.08^*^			
DIM × PH				−0.10^*^	0.04	−2.58^*^
Intercept	2.84	0.27	10.58^***^	3.12	0.24	13.06^***^
*R*^2^	0.23			0.41		
F	7.23^***^			16.42^***^		

*Reported coefficients are unstandardized (with robust standard errors)*.

* *p < 0.05*,

** *p < 0.01*,

*** *p < 0.001*.

**Figure 2:**
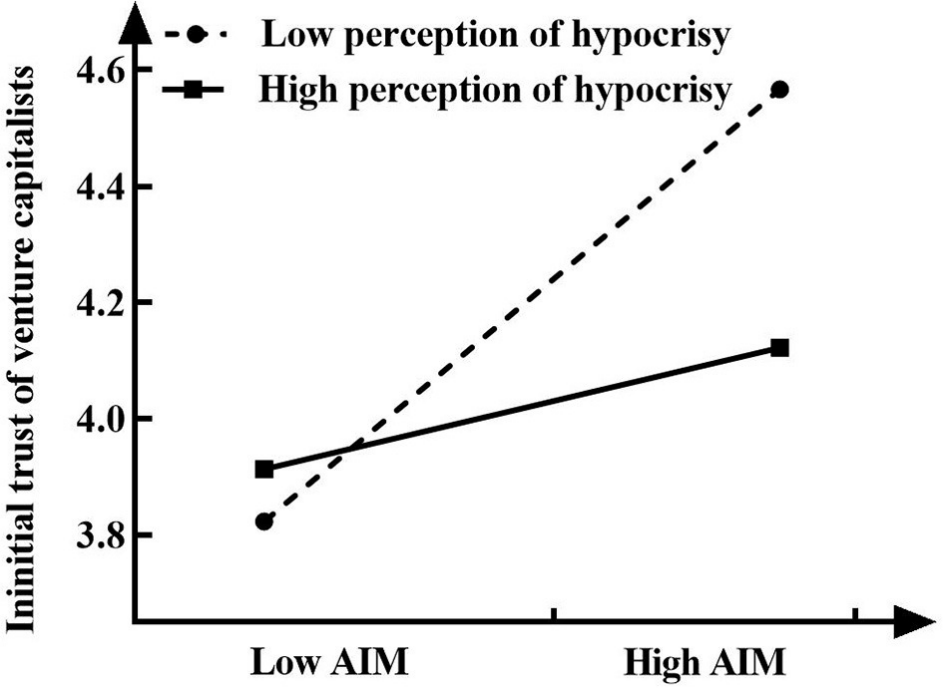
Interactive effect of the acquired impression management strategies and perception of hypocrisy on initial trust of venture capitalists.

**Figure 3:**
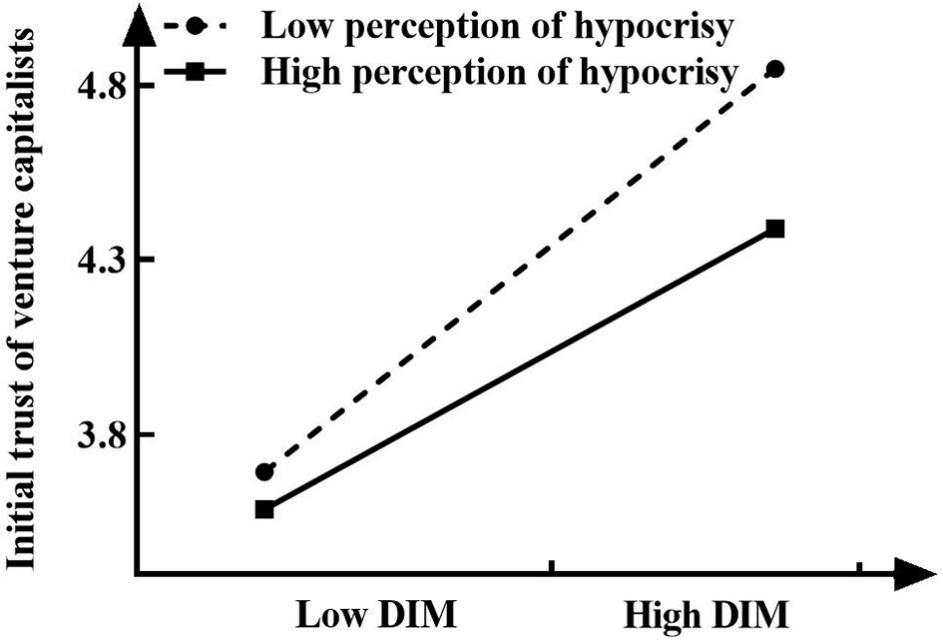
Interactive effect of the defensive impression management strategies and perception of hypocrisy on initial trust of venture capitalists.

To further test whether the moderating effect of perception of hypocrisy is in line with the presupposed expectation, this study follows the methods proposed by Aiken and West (1991) to conduct a further simple slope analysis (see Figures 2, 3). As Figure 2 shows, when the perception of hypocrisy (M-SD) is low, the entrepreneur's use of acquired impression management strategies has a positive effect on the initial trust of venture capitalists (γ = *0.343, t* = *4.239, p* < *0.001*), while when the perception of hypocrisy (M+SD) is high, the entrepreneur's use of acquired impression management strategies no longer has a significant effect on the initial trust of venture capitalists (γ = *0.096, t* = *1.240, p* > *0.05*), which further supports H3a. Figure 3 shows that there is a strong positive correlation between entrepreneurs' defensive impression management strategies and the initial trust of venture capitalists when the perception of hypocrisy (M-SD) is low (γ = *0.693, t* = *8.014, p* < *0.001*). When the perception of hypocrisy (M+SD) is high, although the entrepreneur's defensive impression management strategies also have a positive effect on the initial trust of venture capitalists, the effect is small (γ = *0.482, t* = *6.220, p* < *0.001*). This shows that with the gradual increase in the perception of hypocrisy, the impact of entrepreneurs' defensive impression management strategies on the initial trust of venture capitalists gradually decreases. H3b is therefore verified.

## Discussion

There are three main conclusions in our study. Firstly, entrepreneurial-oriented signals have a positive impact on the initial trust of venture capitalists. This conclusion is consistent with our expected hypothesis, which shows that entrepreneurs can gain the initial trust of venture capitalists by releasing entrepreneurial-oriented signals. Previous studies have shown that entrepreneurs can convey their potential qualities and abilities to venture capitalists through information signals at the stage of entrepreneurial presentation (Eddleston et al., 2014; Ahlers et al., 2015; Wang et al., 2021), to communicate to venture capitalists how committed they are to them through interpersonal signals (Busenitz et al., 2005), and to get along well with others (Huang and Knight, 2015). For example, Ciuchta et al. (2018) found that the entrepreneurial coachability can be used as an interpersonal signal and then affect the investment invention of potential investors. In our research, perseverance orientation as the embodiment of entrepreneurial characteristics can also be used as an interpersonal signal to influence the judgment of venture capitalists on the initial trust of entrepreneurs. In fact, our research not only confirms that adherence to entrepreneurial orientation can be used as interpersonal signals but also verifies that other entrepreneurial orientations such as passion, risk-taking, innovation, and proactivity, can also be used as signals to influence the initial trust of venture capitalists.

Second, entrepreneurs' impression management strategy plays a part of mediating role between entrepreneurial orientation and initial trust of VC. This conclusion is roughly consistent with the conclusion of Tang et al. (2020). They found that community members who pursue knowledge co-creation motivation and online social motivation tend to adopt acquired impression management behavior, while community members who pursue community identity motivation tend to adopt protective impression management behavior. This conclusion shows that individuals will adopt different impression management strategies according to their different goal-oriented motivations. Moreover, our conclusion explains the differences of previous entrepreneurs' use of impression management on resource acquisition. Previous studies have pointed out that Chinese entrepreneurs can obtain support through self-improvement strategies, that is, by showing their abilities and achievements to the audience (Baron and Tang, 2009). However, some studies have found that novice entrepreneurs who use appropriate positive language in their business plans to show their innovation and vulnerability are more likely to gain the support of investors (Parhankangas and Ehrlich, 2014). Based on the different psychological mechanisms of impression management when entrepreneurs transmit entrepreneurship-oriented signals, this study verifies the mediating roles of acquired impression management strategies and defensive impression management in different entrepreneurial orientations and initial trust of venture capitalists. Our results integrate the above differences, which not only further confirm the necessity of refining impression management strategies but also respond to the scholars' appeal to pay more attention to the differences of signal content conveyed by impression management (Yu and Chen, 2019).

Third, perception of hypocrisy negatively moderates the relationship between impression management strategies and the initial trust of VC. Specifically, the lower the VC's perception of hypocrisy on the entrepreneur, the stronger the positive impact of the entrepreneur's acquired impression management strategy and defensive impression management strategy on the initial trust of the venture capitalist, and vice versa. This indicates that when entrepreneurs convey different entrepreneurial orientation signals to venture capitalists through different impression management strategies, they should not only take into account the need to actively show themselves to venture capitalists but also reduce the concerns of venture capitalists about their own integrity and ability. Otherwise, once the venture capitalist perceives the entrepreneur to be hypocritical, his or her initial trust will be greatly reduced. As founded by Chen and Ye (2010), behavioral consistency is an important criterion for venture capitalists to assess the credibility of entrepreneurs. In addition, this conclusion is similar to the previous research conclusions. The perception of entrepreneurial behavioral consistency is positively correlated with venture capitalists' investment decisions (Yang and Li, 2018).

### Theoretical Contributions

The theoretical contributions of this paper are shown in the following three main aspects. First, our study links the entrepreneurial orientation with the initial trust of venture capitalists and expands the research about the antecedent variable of the initial trust of venture capitalists. Previous studies have theoretically emphasized that the initial trust of venture capitalists on entrepreneurs before cooperation is characterized by competence, goodwill, and integrity and pointed out that this trust helps to promote the initial investment of venture capitalists, thus affecting mutual trust after investment (Wang and Dong, 2018). Although a few scholars have studied the initial trust, they mainly based on case analysis to discuss how the trust relationship between the parties evolves after cooperation, and there are few studies on the influencing factors of the initial trust before the stage of cooperation. As a supplement, based on the signal theory, this study starts from the “relationship situation before cooperation” and examines the influence of different entrepreneurial orientations on the initial trust of venture capitalists. The conclusions reveal the influence of different entrepreneurial orientations on the initial trust of venture capitalists, which not only verifies the signal function of entrepreneurial orientation but also provides a supplement for the research on individual entrepreneurial orientation and expands the antecedent variables of initial trust of venture capitalists.

Second, we propose and test the mediating role of impression management strategies between entrepreneurial orientation and initial trust and refine the legitimacy of impression management. Previous studies have mostly explained the construction of trust between venture capitalists and entrepreneurs based on the perspectives of resource conservation theory, social exchange theory, and social relationship networks, while there are relatively few studies from the perspective of impression management strategy theory. In the existing research on the role of impression management, most of the research has discussed the effects of impression management, such as how entrepreneurs use impression management to gain or maintain legitimacy, especially to resist stigma and obtain entrepreneurial well-being after failure. However, few scholars have paid attention to entrepreneurs' impression management strategies and venture capitalists' initial trust before the establishment of entrepreneurial partnerships. This study discusses and confirms that the initial trust of entrepreneurial orientation to venture capitalists is mediated by the entrepreneur's impression management strategy. The introduction of the impression management strategy not only reveals the psychological mechanism of entrepreneurial orientation as a signal to the initial trust of venture capitalists but also breaks through the phenomenon that the previous literature mainly uses resource preservation and social exchange as the formation mechanism of trust.

Third, this study explains under what conditions different impression management strategies affect the initial trust of venture capitalists. Although entrepreneurs can transmit different entrepreneurial orientation signals to venture capitalists by using impression management strategies, and they will get the initial trust of venture capitalists, but venture capitalists will not blindly trust them, which means that whether different impression management strategies can ultimately transform the initial trust of venture capitalists depends on the cognitive evaluation of venture capitalists. Previous studies have confirmed that when venture capitalists make investment decisions, the consistency of entrepreneurial behavior is an important criterion for venture capitalists to assess their credibility. In addition, previous scholars have found that the consistency of individual behavior has an impact on the decision-making of venture capitalists. However, the current research has not examined the boundary conditions of impression management and initial trust of venture capitalists. Therefore, this study extends the existing literature on the influence of impression management strategies on initial trust boundaries.

### Practical Implications

The conclusions of this study provide some practical enlightenment for VC-E on how to establish the cooperative relationship of initial trust. Firstly, in the process of venture financing, entrepreneurs must improve the initial trust perception of venture capitalists. Previous studies have shown that the trust of investors can reduce the cost of transactions between the two parties, which is a generalization of fait accomplished. However, the perception of investor trusts is a keen grasp of the external investment environment of entrepreneurship, which is an ex-ante perception. Only by enhancing the initial trust of venture capitalists can we better promote the transactions and cooperation between the two parties. The different entrepreneurial orientation of entrepreneurs can be used as a signal to promote the initial trust of venture capitalists. For this reason, entrepreneurs need to actively shape their own entrepreneurial orientation in the process of starting a business. Entrepreneurs should pay more attention to the continuous changes of the external entrepreneurial environment, have the spirit of risk-taking, dare to innovate, pursue excellence, pay attention to external knowledge learning, strive to improve their business ability, deal with the difficulties in the entrepreneurial process with full entrepreneurial passion, and persevere. Only in this way can he or she describe to venture capitalists that start-ups have better development potential and positive signals of profitability.

Next, entrepreneurs should be aware of the difference between the role of impression management strategies in entrepreneurial orientation and initial trust of venture capitalists. In order to reduce the risk investment in trust, venture capitalists always need as much information as possible as decision-making basis when they evaluate the trust of entrepreneurs. In the face of fierce external market competition and high-speed market reaction demand, how to make ideal investment among potential entrepreneurs with low cost and high return needs a high initial trust. Entrepreneurs should be more rational about the driving effect of impression management strategy on the initial trust of venture capitalists. On the one hand, entrepreneurs should actively reveal their own abilities and characteristics; on the other hand, they should also pay attention to the differences in this way of expression. Only reasonable use of impression management strategy can give play to its effectiveness and avoid too low impression management or too high impression management, which causes venture capitalists to question their ability and integrity.

Finally, entrepreneurs need to maintain their own behavior consistency in order to reduce the negative impact of perception of hypocrisy. In the initial relationship, there is no empirical knowledge to distinguish the belief of the trusted party from the belief of the environment, and the cognitive consistency is particularly prominent. Faced with the asymmetry of information, when entrepreneurs deliver credible information to venture capitalists through active verbal communication and specific behaviors, they should reduce the uncertainty of this information, maintain the consistency of their own words and actions, and let venture capitalists perceive their sincerity, making them make more benevolent attributions, thereby promoting initial trust. Otherwise, the excessively high perception of hypocrisy will make the effect of the impression management strategy backfire.

### Research Limitations and Future Research Directions

There are some limitations to this study. First, the research variable data sample is self-reported by venture capitalists. Although there is no serious common method deviation problem, it still exists objectively. In future research, we will consider using questionnaires from both venture capitalists and entrepreneurs for data collection. Moreover, this study analyzes the mediating effect of impression management strategies based only on impression management theory, and subsequent research can adopt other theories to further explore the mechanism. For example, future studies can combine the contextual elements of “guanxi” in China to explore the factors forming trust in entrepreneurs by venture capitalists at different stages and their impact on investment decisions. Finally, the perception of hypocrisy may not be the only boundary condition that affects the relationship between impression management strategies and the initial trust of venture capitalists. Scholars can also discuss the effectiveness of entrepreneurs in adopting acquired impression management strategies or defensive impression management strategies under specific circumstances.

## Conclusion

Start-ups are at the disadvantage of being “new and weak;” in the early stage of entrepreneurship, the lack of resources restricts their development. How to obtain the support from external resources becomes the main problem for entrepreneurs. In the critical stage of resource acquisition, venture capitalists' initial trust in entrepreneurs plays an important role. High initial trust is conducive to rationally dealing with the emotional relationships about the two parties and to effectively promoting the financing of entrepreneurial projects. The effectiveness of trust has been extensively investigated in entrepreneurship studies. However, compared to the outcomes of trust, it is rare for previous researches to focus on the mechanisms underlying venture capitalists' initial trust in entrepreneurs. Based on signal theory and impression management theory, the study examines the influence mechanism and boundary conditions of different entrepreneurial orientations on the initial trust of venture capitalists.

The present study has investigated how different entrepreneurial orientation improves venture capitalists' initial trust through entrepreneurs' impression management strategies. We have found that entrepreneurs' perseverance orientation and passion orientation have an indirect positive effect on the initial trust of venture capitalists through the mediating effect of acquired impression management strategies, while risk-taking, innovation, and proactivity orientation are mediated by defensive impression management strategies. In addition, the perception of hypocrisy not only moderates the relationship between acquired impression management strategies and the initial trust of venture capitalists but also moderates the relationship between defensive impression management strategies and the initial trust of venture capitalists. When the venture capitalist has low perceptions of hypocrisy, the positive effect of acquired and defensive impression management strategies on the initial trust becomes strengthened. We hope this study will enhance our current knowledge on the venture capitalists' initial trust in entrepreneurs and provide some new insights.

## Data Availability Statement

The raw data supporting the conclusions of this article will be made available by the authors, without undue reservation.

## Ethics Statement

Ethical review and approval was not required for the study on human participants in accordance with the local legislation and institutional requirements. The patients/participants provided their written informed consent to participate in this study.

## Author Contributions

HY, LZ, and YW conceived and supervised the study. HY and LZ wrote the manuscript. HS analyzed the data. YW and SX improved the manuscript. All authors contributed equally to this manuscript and reviewed and approved this manuscript for publication.

## Conflict of Interest

The authors declare that the research was conducted in the absence of any commercial or financial relationships that could be construed as a potential conflict of interest.

## References

[B1] Ahlers G. Cumming D. Guenther C. Schweizer D. (2015). Signaling in equity crowdfunding. Entrep. Theory Pract. 39, 955–980. 10.1111/etap.12157

[B2] Aiken L. S. West S. G. (1991). Multiple Regression: Testing and Interpreting Interactions. London: Sage Publications.

[B3] Aldrich H. E. Fiol C. M. (1994). Fools rush in? The institutional context of industry creation. Acad. Manag. Rev. 19, 645–670. 10.5465/amr.1994.9412190214

[B4] Alvarez S. A. Busenitz L. W. (2001). The entrepreneurship of resource-based theory. J. Manag. 27, 755–775. 10.1177/014920630102700609

[B5] Anglin A. H. Wolfe M. T. Short J. C. McKenny A. F. Pidduck R. J. (2018). Narcissistic rhetoric and crowdfunding performance: a social role theory perspective. J. Bus. Ventur. 33, 780–812. 10.1016/j.jbusvent.2018.04.004

[B6] Ashforth B. E. Gibbs B. W. (1990). The double-edge of organizational legitimation. Organ. Sci. 1, 177–194. 10.1287/orsc.1.2.177

[B7] Baron R. A. Tang J. (2009). Entrepreneurs' social skills and new venture performance: mediating mechanisms and cultural generality. J. Manag. 35, 282–306. 10.1177/0149206307312513

[B8] Bolino M. C. Kacmar K. M. Turnley W. H. Gilstrap J. B. (2008). A multi-level review of impression management motives and behaviors. J Manag. 34, 1080–1109. 10.1177/0149206308324325

[B9] Busenitz L. W. Fiet J. O. Moesel D. D. (2005). Signaling in venture capitalist-New venture team funding decisions: does it indicate long-term venture outcomes. Entrep. Theory Pract. 29, 1–12. 10.1111/j.1540-6520.2005.00066.x

[B10] Cardon M. S. Gregoire D. A. Stevens C. E. Patel P. C. (2013). Measuring entrepreneurial passion: conceptual foundations and scale validation. J. Bus. Ventur. 28, 373–396. 10.1016/j.jbusvent.2012.03.003

[B11] Cardon M. S. Wincent J. Singh J. Drnovsek M. (2009). The nature and experience of entrepreneurial passion. Acad. Manag. Rev. 34, 511–532. 10.5465/amr.2009.40633190

[B12] Chen C. Ye Y. (2010). Influence factor asymmetry of VC-E trust: a multi-case study. China Ind. Eco 1, 147–157. 10.19581/j.cnki.ciejournal.2010.01.014

[B13] Chen X. P. Yao X. Kotha S. (2009). Entrepreneur passion and preparedness in business plan presentations: a persuasion analysis of venture capitalists' funding decisions. Acad. Manage. J. 52, 199–214. 10.5465/amj.2009.36462018

[B14] Cholakova M. Clarysse B. (2015). Does the possibility to make equity investments in crowdfunding projects crowd out reward–based investments? Entrep. Theory Pract. 39, 145–172. 10.1111/etap.12139

[B15] Ciuchta M. P. Letwin C. Stevenson R. McMahon S. Huvaj M. N. (2018). Betting on the coachable entrepreneur: signaling and social exchange in entrepreneurial pitches. Entrep. Theory Pract. 42, 860–885. 10.1177/1042258717725520

[B16] Connelly B. L. Certo S. T. Ireland R. D. Reutzel C. R. (2011). Signaling theory: a review and assessment. J. Manag. 37, 39–67. 10.1177/0149206310388419

[B17] Covin J. G. Lumpkin G. T. (2011). Entrepreneurial orientation theory and research: reflections on a needed construct. Entrep. Theory Pract. 35, 855–872. 10.1111/j.1540-6520.2011.00482.x

[B18] Covin J. G. Slevin D. P. (1989). Strategic management of small firms in hostile and benign environments. Strateg. Manag. J. 10, 75–87. 10.1002/smj.4250100107

[B19] Covin J. G. Wales W. J. (2012). The measurement of entrepreneurial orientation. Entrep. Theory Pract. 36, 677–702. 10.1111/j.1540-6520.2010.00432.x

[B20] Davis B. C. Hmieleski K. M. Webb J. W. Coombs J. E. (2017). Funders' positive affective reactions to entrepreneurs' crowdfunding pitches: the influence of perceived product creativity and entrepreneurial passion. J. Bus. Ventur. 32, 90–106. 10.1016/j.jbusvent.2016.10.006

[B21] Deng S. Cui L. Gu C. Yan X. (2020). Entrepreneurs' goal orientation and effectuation: a moderated mediation model. R D Manag. 10.13581/j.cnki.rdm.20191475. [Epub ahead of print].

[B22] Dimotakis N. Conlon D. E. Ilies R. (2012). The mind and heart (literally) of the negotiator: personality and contextual determinants of experiential reactions and economic outcomes in negotiation. J. Appl. Psychol. 97, 183–193. 10.1037/a0025706 21967294

[B23] Donbesuur F. Boso N. Hultman M. (2020). The effect of entrepreneurial orientation on new venture performance: contingency roles of entrepreneurial actions. J. Bus. Res. 118, 150–161. 10.1016/j.jbusres.2020.06.042

[B24] Eddleston K. A. Ladge J. J. Mitteness C. Balachandra L. (2014). Do you see what I see? signaling effects of gender and firm characteristics on financing entrepreneurial ventures. Entrep. Theory Pract. 40, 489–514. 10.1111/etap.12117

[B25] Effron D. A. O'Connor K. Leroy H. Lucas B. J. (2018). From inconsistency to hypocrisy: when does “saying one thing but doing another” invite condemnation. Res. Organ. Behav. 38, 61–75. 10.1016/j.riob.2018.10.003

[B26] Eisenberger R. Leonard J. M. (1980). Effects of conceptual task difficulty on generalized persistence. Am. J. Psychol. 93, 285–298. 10.2307/1422233 7406069

[B27] Elsbach K. D. Sutton R. I. (1992). Acquiring organizational legitimacy through illegitimate actions: a marriage of institutional and impression management theories. Acad. Manag. J. 35, 699–738. 10.2307/256313

[B28] Fisher G. Kuratko D. F. Bloodgood J. M. Hornsby J. S. (2017). Legitimate to whom? the challenge of audience diversity and new venture legitimacy. J. Bus. Ventur. 32, 52–71. 10.1016/j.jbusvent.2016.10.005

[B29] Gao Y. Zhang D. Ma H. Du X. (2020). Exploring creative entrepreneurs' IEO: extraversion, neuroticism, and creativity. Front. Psychol. 11:2170. 10.3389/fpsyg.2020.02170 32982883 PMC7485561

[B30] Gerschewski S. Lindsay V. J. Rose E. (2016). Advancing the entrepreneurial orientation construct: the role of passion and perseverance. Rev. Int. Bus. Strategy 26, 446–471. 10.1108/RIBS-08-2016-0042

[B31] Granqvist N. Grodal S. Woolley J. L. (2013). Hedging your bets: explaining executives' market labeling strategies in nanotechnology. Organ. Sci. 24, 395–413. 10.1287/orsc.1120.0748

[B32] Greenbaum R. L. Mawritz M. B. Piccolo R. F. (2015). When leaders fail to “walk the talk” supervisor undermining and perceptions of leader hypocrisy. J. Manag. 41, 929–956. 10.1177/0149206312442386

[B33] Gupta V. Gupta A. (2015). The concept of entrepreneurial orientation. Found. Trends Entrepreneurship 11, 55–137. 10.1561/0300000054

[B34] Hayes A. F. (2013). Introduction to Mediation, Moderation, and Conditional Process Analysis: A Regression-Based Approach. New York, NY: Guilford publications.

[B35] He P. Zhou Q. Zhao H. Jiang C. Wu Y. J. (2020). Compulsory citizenship behavior and employee creativity: creative self-efficacy as a mediator and negative affect as a moderator. Front. Psychol. 11:1640. 10.3389/fpsyg.2020.01640 32793046 PMC7385136

[B36] Hirst G. Van Knippenberg D. Zhou J. (2009). A cross-level perspective on employee creativity: goal orientation, team learning behavior, and individual creativity. Acad. Manag. J. 52, 280–293. 10.5465/amj.2009.37308035

[B37] Hu R. Wang L. Zhang W. Bin P. (2018). Creativity, proactive personality, and entrepreneurial intention: the role of entrepreneurial alertness. Front. Psychol. 9:951. 10.3389/fpsyg.2018.00951 29962985 PMC6011088

[B38] Huang L. Knight A. (2015). Resources and relationships in entrepreneurship: an exchange theory of the development and effects of the entrepreneur-investor relationship. Acad. Manag. Rev. 42, 80–102. 10.5465/amr.2014.0397

[B39] Huang Y. Wilkinson I. F. (2013). The dynamics and evolution of trust in business relationships. Ind. Mark. Manag. 42, 455–465. 10.1016/j.indmarman.2013.02.016

[B40] Kibler E. Mandl C. Kautonen T. Berger E. S. (2017). Attributes of legitimate venture failure impressions. J. Bus. Ventur. 32, 145–161. 10.1016/j.jbusvent.2017.01.003

[B41] Koe W. L. (2016). The relationship between Individual Entrepreneurial Orientation (IEO) and entrepreneurial intention. J. Glob. Entrep. Res. 6:13. 10.1186/s40497-016-0057-8

[B42] Langkamp Bolton D. Lane M. D. (2012). Individual entrepreneurial orientation: development of a measurement instrument. Educ. Train. 54, 219–233. 10.1108/00400911211210314

[B43] Leary M. R. Kowalski R. M. (1990). Impression management: a literature review and two-component model. Psychol. Bull. 107, 34–47. 10.1037/0033-2909.107.1.34

[B44] Li C. Zhu Y. Wang Y. (2018). A study of the influence mechanism of entrepreneurial passion on entrepreneurial persistence. Sci Res Manag. 9, 134–142. 10.19571/j.cnki.1000-2995.2018.09.015

[B45] Li Y. Zhao W. Xu C. (2018). Research on the relationship between entrepreneurial orientation, social network, and knowledge resources acquisition: signaling theory perspective. Sci. Manag. S T 39, 130–141.

[B46] Liu X. Lin C. Zhao G. Zhao D. (2019). Research on the effects of entrepreneurial education and entrepreneurial self-efficacy on college students' entrepreneurial intention. Front. Psychol. 10:869. 10.3389/fpsyg.2019.00869 31068862 PMC6491517

[B47] Lumpkin G. Dess G. (1996). Clarifying the entrepreneurial orientation construct and linking it to performance. Acad. Manag. Rev. 21, 135–172. 10.5465/amr.1996.9602161568

[B48] Ma X. Yan S. (2016). Study on the Mechanism of entrepreneurial orientation to organizational creativity—from the perspective of organizational context. R D Manag. 28, 73–83. 10.13581/j.cnki.rdm.2016.01.007

[B49] MacKinnon D. P. (2008). Introduction to Statistical Mediation Analysis. New York, NY: Taylor and Francis Group Publications.

[B50] Maula M. V. Keil T. Zahra S. A. (2013). Top management's attention to discontinuous technological change: corporate venture capital as an alert mechanism. Organ. Sci. 24, 926–947. 10.1287/orsc.1120.0775

[B51] Maxwell A. L. Jeffrey S. A. Lévesque M. (2011). Business angel early stage decision making. J. Bus.Venture. 26, 212–225. 10.1016/j.jbusvent.2009.09.002

[B52] McDougall P. Robinson R. B. Jr. (1990). New venture strategies: an empirical identification of eight “archetypes” of competitive strategies for entry. Strateg. Manag. J. 11, 447–467. 10.1002/smj.4250110604

[B53] McKnight D. H. Choudhury V. Kacmar C. (2002). The impact of initial consumer trust on intentions to transact with a web site: a trust building model. J. Strateg. Inf. Syst. 11, 297–323. 10.1016/S0963-8687(02)00020-3

[B54] Mitteness C. R. Baucus M. S. Sudek R. (2012). Horse vs. jockey? how stage of funding process and industry experience affect the evaluations of angel investors. Ventur. Cap. 14, 241–267. 10.1080/13691066.2012.689474

[B55] Mollick E. (2014). The dynamics of crowdfunding: an exploratory study. J. Bus. Ventur. 29, 1–16. 10.1016/j.jbusvent.2013.06.005

[B56] Moss T. W. Neubaum D. O. Meyskens M. (2015). The effect of virtuous and entrepreneurial orientations on microfinance lending and repayment: a signaling theory perspective. Entrep. Theory Pract. 39, 27–52. 10.1111/etap.12110

[B57] Nagy B. G. Pollack J. M. Rutherford M. W. Lohrke F. T. (2012). The influence of entrepreneurs' credentials and impression management behaviors on perceptions of new venture legitimacy. Entrep. Theory Pract. 36, 941–965. 10.1111/j.1540-6520.2012.00539.x

[B58] Parhankangas A. Ehrlich M. (2014). How entrepreneurs seduce business angels: an impression management approach. J. Bus. Ventur. 29, 543–564. 10.1016/j.jbusvent.2013.08.001

[B59] Park H. D. Steensma H. K. (2012). When does corporate venture capital add value for new ventures? Strateg. Manag. J. 33, 1–22. 10.1002/smj.937

[B60] Pollack J. M. Bosse D. A. (2014). When do investors forgive entrepreneurs for lying. J. Bus. Ventur. 29, 741–754. 10.1016/j.jbusvent.2013.08.005

[B61] Qin F. Wright M. Gao J. (2017). Are “sea turtles” slower? returnee entrepreneurs, venture resources, and speed of entrepreneurial entry. J. Bus. Ventur. 32, 694–706. 10.1016/j.jbusvent.2017.08.003

[B62] Rahim A. W. P. A. Ismail W. K. W. Thurasamy R. Abd I. (2018). The relationship of individual creativity with entrepreneurial intention via individual entrepreneurial orientation (IEO). Int. J. Innov. Bus. Strategy 9, 41–54.

[B63] Rousseau D. M. Sitkin S. B. Burt R. S. Camerer C. (1998). Not so different after all: a cross-discipline view of trust. Acad. Manag. Rev. 23, 393–404. 10.5465/amr.1998.926617

[B64] Santos G. Marques C. S. Ferreira J. J. (2020). Passion and perseverance as two new dimensions of an individual entrepreneurial orientation scale. J. Bus. Res. 112, 190–199. 10.1016/j.jbusres.2020.03.016

[B65] Schoorman F. D. Mayer R. C. Davis J. H. (2007). An integrative model of organizational trust: past, present, and future. Acad. Manag. Rev. 32, 344–354. 10.5465/amr.2007.24348410

[B66] Shan P. Song M. Ju X. (2016). Entrepreneurial orientation and performance: is innovation speed a missing link. J. Bus. Res. 69, 683–690. 10.1016/j.jbusres.2015.08.032

[B67] Shane S. Locke E. A. Collins C. J. (2003). Entrepreneurial motivation. Hum. Resour. Manag. Rev. 13, 257–279. 10.1016/S1053-4822(03)00017-2

[B68] Si X. Wang S. Fu Y. (2016). Where do entrepreneurial opportunities come from: discovery, construction, or discovery and construction? the frontier research on the theory of entrepreneurial opportunity. Mana. World 3, 115–127. 10.19744/j.cnki.11-1235/f.2016.03.010

[B69] Tang X. Zhou P. Su H. (2020). A perspective of on-line impression management: a study of the relationship between innovation motivation and innovation performance. Sci. Res. Manag. 41, 172–180. 10.19571/j.cnki.1000-2995.2020.06.018

[B70] Uy M. A. Sun S. Foo M. D. (2017). Affect spin, entrepreneurs' well-being, and venture goal progress: the moderating role of goal orientation. J. Bus. Ventur. 32, 443–460. 10.1016/j.jbusvent.2016.12.001

[B71] Von Gehlen K. Holtgrave M. Nienaber A.-M. Schewe G. (2018). Trust in entrepreneur-venture capitalist relationships: a bilateral perspective, in 2018 Portland International Conference on Management of Engineering and Technology (PICMET) (Honolulu, HI).

[B72] Wales W. J. Patel P. C. Parida V. Kreiser P. M. (2013). Nonlinear effects of entrepreneurial orientation on small firm performance: the moderating role of resource orchestration capabilities. Strateg. Entrepreneurship J. 7, 93–121. 10.1002/sej.1153

[B73] Wang W. Chen W. Zhu X. Wang H. (2016). The impact of Linguistic persuasiveness on the success rate of crowdfunding campaigns: evidence from Kickstarter. Manag. World 5, 81–98. 10.19744/j.cnki.11-1235/f.2016.05.008

[B74] Wang W. He L. Wu Y. J. Goh M. (2021). Signaling persuasion in crowdfunding entrepreneurial narratives: the subjectivity vs objectivity debate. Comput. Hum. Behav. 114:106576. 10.1016/j.chb.2020.106576

[B75] Wang X. Dong N. (2018). How trust is built and evolves in the context of chinese crowdfunding? a case study of Shui Mu Ke's crowdfunding. Bus. Rev. 30, 242–255. 10.14120/j.cnki.cn11-5057/f.2018.01.023

[B76] Ward T. B. (2004). Cognition, creativity, and entrepreneurship. J. Bus. Ventur. 19, 173–188. 10.1016/S0883-9026(03)00005-3

[B77] Wei H. Long L. (2008). Interpersonal initial trust within organizations. Adv. Psychol. Sci. 16, 328–334. Available online at: http://journal.psych.ac.cn/xlkxjz/EN/Y2008/V16/I02/328

[B78] Wu Y. Wu T. Sharpe J. (2020). Consensus in the definition of social entrepreneurship: a content analysis approach. Manag. Decis. 58, 2593–2619. 10.1108/MD-11-2016-0791

[B79] Yang H. Li H. (2017). Trust cognition of entrepreneurs' behavioral consistency modulates investment decisions of venture capitalists in cooperation. Entrepreneurship Res. J. 8, 1–15. 10.1515/erj-2017-0084

[B80] Yang H. Li H. (2018). Research on dynamic factors of venture capitalists' trust on entrepreneurs based on warshall-anp. Sci. Technol. Prog. Policy 35, 129–136. 10.6049/kjjbydc.2017080415

[B81] Yao N. Zhang Y. Zhou F. (2019). Impact of self-sacrificial leadership on employee voice: a moderated mediation model. Sci. Res. Manag. 40, 221–230. 10.19571/j.cnki.1000-2995.2019.09.022

[B82] Yi C. Zhou F. (2011). A research on entrepreneurial performance of venture firm: from the respective of trust between entrepreneur and venture capitalist. Stud. Sci. 29, 591–600+609. 10.16192/j.cnki.1003-2053.2011.04.016

[B83] Yi C. H. (2011). Empirical study on the structure model of trust between entrepreneur and venture capitalist. Stud Sci Sci.29, 914–923. 10.16192/j.cnki.1003-2053.2011.06.017

[B84] Yu X. Chen Y. (2019). A literature review on impression management in entrepreneurship and future prospects. Chin. J. Manag. 16, 1255–1264. 10.3969/j.issn.1672-884x.2019.08.017

[B85] Zhang X. Li M. (2019). Research on the influence of social support on entrepreneurial persistence. Stud. Sci. 37, 2008–2015. 10.16192/j.cnki.1003-2053.2019.11.010

[B86] Zhang X. Li M. (2020). Research on the driving factors of entrepreneurial resilience and its influence on entrepreneurial success. Fore Econ. Manag. 42, 96–108. 10.16538/j.cnki.fem.20200519.401

[B87] Zhao B. Zhao Y. (2019). Study on the effect of impression management motivation on employees' innovation behavior. Sci. Technol. Prog. Policy 36, 145–153. 10.6049/kjjbydc.2018060027

